# Chronic developmental hypoxia alters rat lung immune cell transcriptomes during allergic airway inflammation

**DOI:** 10.14814/phy2.15600

**Published:** 2023-02-07

**Authors:** Michelle Chu, Huanling Gao, Patricia Esparza, Abigail Pajulas, Jocelyn Wang, Rakshin Kharwadkar, Hongyu Gao, Mark H. Kaplan, Robert S. Tepper

**Affiliations:** ^1^ Department of Microbiology and Immunology Indiana University Indianapolis Indiana USA; ^2^ Department of Pediatrics and Herman B Wells Center for Pediatric Research Indiana University Indianapolis Indiana USA; ^3^ Department of Medical and Molecular Genetics Indiana University Indianapolis Indiana USA

**Keywords:** airway inflammation, hypoxia, single‐cell RNA sequencing

## Abstract

Populations that are born and raised at high altitude develop under conditions of chronic developmental hypoxia (CDH), which results in pulmonary adaptations of increased lung volume and diffusion capacity to increase gas exchange. It is not clear how CDH may alter allergic inflammation in the lung. In this study, we sought to characterize the impact of CDH on immune cell populations in the rat lung during a murine model of asthma. Rats were bred and raised in either hypoxic (15% oxygen, CDH) or normobaric room air (20% oxygen). At 3‐weeks of age, animals were sensitized to ovalbumin (OVA) or physiologic saline (phosphate‐buffered saline [PBS]) as a control, followed by three consecutive days of intra‐nasal OVA or PBS at 6‐weeks of age. We then assessed airway reactivity and allergic‐associated cytokine levels. This was followed by single‐cell transcriptomic profiling of lung cell populations. In scRNA‐seq analysis, we assessed differentially expressed genes, differentially enriched functional pathways, immune cell exhaustion/activation markers, and immune cell secretory products. Our results show that while OVA heightened airway reactivity, CDH suppressed airway reactivity in OVA‐challenged and control animals. Through scRNA‐seq analysis, we further demonstrate that CDH alters the transcriptional landscape in the lung and alters transcriptional programs in immune cells. These data define CDH‐dependent changes in the lung that impact airway reactivity.

## Graphical abstract

Populations that are born and raised at high altitude develop under conditions of chronic developmental hypoxia (CDH), which results in pulmonary adaptations of increased lung volume and diffusion capacity to increase gas exchange. It is not clear how CDH may alter allergic inflammation in the lung. In this study, we defined the impact of CDH on airway hyperreactivity and immune cell populations in the rat lung during a model of asthma.

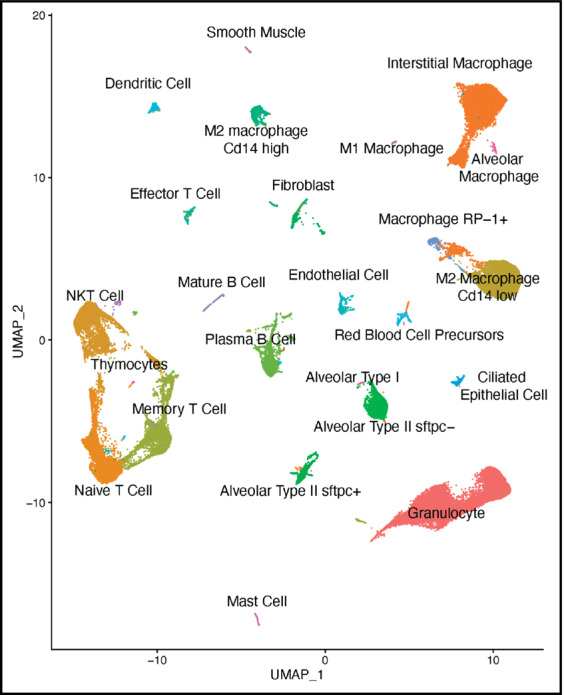

## 1 INTRODUCTION

Over 140 million people worldwide live at an altitude higher than 2500 m above sea level. Populations that live at high altitude develop and thrive in a chronic hypoxic state (Frisancho et al., 1997). To acclimate to hypoxia, residents at high altitudes must make physiological adaptions to increase oxygen transport to tissues or alter cellular metabolism. We have previously shown that newborns at high altitudes have increased lung volumes compared to infants at lower altitude, an adaptation that allows for increased oxygen exchange across the larger surface area (Frisancho et al., 1995). Additionally, this adaptation is maintained in older children and adults who experienced chronic hypoxia since birth (DeGraff et al., 1970; Droma et al., 1991; Greksa et al., 1994; Guleria et al., 1971; Mueller et al., 1978; Remmers & Mithoefer, 1969).

Physiological alterations in lung structure and function under hypoxic conditions alongside the qualitative observation that patients with allergic airway diseases experience attenuation of symptoms when residing in high mountainous regions suggested that hypoxic environments might improve asthmatic patient outcomes. One confounding aspect of this phenomenon is the fact that there is decreased abundance of common allergens, such as house dust mite, at high altitudes which then naturally leads to decreased exacerbation of asthmatic symptoms (Spieksma et al., 1971; van Velzen et al., 1996). However, the lower allergen titers do not fully explain the decrease in asthmatic symptoms at high altitudes. Rijssenbeek‐Nouwens et al. (2012) have shown that non‐allergic patients with severe refractory asthma also demonstrate decreased airway inflammation in response to high altitude conditions indicating that hypoxic conditions have potential direct effects on the asthmatic response.

Hypoxia induces expression of HIF‐1α (hypoxia inducible factor 1 subunit alpha), a transcription factor that is a master regulator of oxygen homeostasis by modulating physiological functions to increase oxygen transport (Semenza, 2001). HIF‐1α activation increases expression of vascular endothelial growth factor to facilitate angiogenesis in response to hypoxic conditions (Forsythe et al., 1996; Liu et al., 1995) and also shifts cells away from cellular respiration and toward oxygen‐independent glycolysis (Cheng et al., 2014; Sun et al., 2020). Additionally, HIF‐1α plays an active role in various immune cell populations. Inhibition of *Hif1a* leads to increased NK cell activity (Ni et al., 2020), decreased dendritic cell glycolysis and migration (Liu et al., 2019), while HIF‐1α induction increased expression of the exhaustion marker Tim‐3 on microglia (Koh et al., 2015). Moreover, several studies have shown that hypoxia promotes T cell exhaustion in the tumor microenvironment. T cells isolated from human PBMCs cultured under hypoxic conditions demonstrated increased *Hif1a* expression, decreased T cell expansion, and increased exhaustion markers (Liu et al., 2020). Hypoxia inhibits effector CD4+ T cell expansion and function in a HIF‐1α‐dependent manner (Westendorf et al., 2017). While the attenuation of immune cell function is not ideal in the context of tumor biology, it is beneficial in the context of allergic airway diseases and several reports demonstrate that hypoxic conditions achieved by high altitude therapy improves lung function and decreases the inflammatory phenotype in asthmatic patients. A meta‐analysis by Vinnikov et al. showed that high altitude climate therapy (HACT) significantly improved lung function in data aggregated from both asthmatic children and adults (Vinnikov et al., 2016). Additionally, Karagiannidis et al. (2006) showed that HACT impacts CD4+ helper T cells decreasing the number of inflammatory, circulating Th2 cells, and decreasing mRNA levels of IFNγ and IL‐13. While these and other studies have investigated the effect of post‐natal hypoxia exposure on lung function and immune cell function, it remains unknown how hypoxia beginning at conception and maintained during the post‐natal development period affects airway reactivity and lung immune cell populations. In this study, we utilized a rat model of chronic developmental hypoxia (CDH) superimposed with a model of allergic airway inflammation to evaluate the effect of CDH on critical asthmatic phenotypes: airway reactivity and lung inflammation. We hypothesize that CDH will decrease airway reactivity, decrease lung inflammation, and alter lung immune cell populations.

## 2 METHODS

### 2.1 Animals and ethical statement

Sprague Dawley rats from Harlan Indianapolis were used in these studies. Studies involving animals in this report were approved by the Institutional Animal Care and Use Committee.

### 2.2 Chronic hypoxia asthma model

Rat breeding pairs were acclimated to either hypoxic (15% FiO_2_) (CDH) or normoxic/room air (RA) conditions for 3 weeks. Pups born to the breeding pairs were then weaned and raised in the same conditions in which they were born. At 4–6 weeks of age, both hypoxic and normoxic animals received intraperitoneal ovalbumin (OVA, 250 μg in phosphate‐buffered saline [PBS]) and pertussis toxin (50 ng) followed by a booster 8–12 days later. Both hypoxic and normoxic rats were then challenged with either intra‐nasal OVA or a PBS vehicle control for 3 days.

### 2.3 Splenocyte stimulation

Spleens of male and female rats were smashed between glass slides and subsequently filtered through a 70 μM filter to dissociated into single cells. Red blood cells were lysed with ACK lysis for 5 min. Splenic cells were then restimulated for 3 days with OVA (250 μg/ml) in RPMI + 1% fetal bovine serum.

### 2.4 scRNA‐seq and analysis

Lung samples were processed individually without pooling. Lungs of male mice were cut into small pieces and incubated with 0.5 mg/ml Collagenase A for 45 min at 37C in 15 ml Dulbecco's Modified Eagle Medium (DMEM). The digested lung was then dissociated into a single‐cell suspension by separation through a 70 μM filter twice. Red blood cells and dead cells were removed with Miltenyi Dead Cell Removal Kit. Cells were resuspended at 1 million/ml and sent to the core for sequencing. Cell viability was assessed at 86%–96% for all samples prior to sequencing. Viability was assessed with Trypan Blue staining followed by counting via hemocytometer. Library preparation for scRNA‐sequencing was performed using following kits: Chromium™ Single Cell 3′ Library and Gel Bead Kit V3, 4 rxns PN‐120267, Chromium Single Cell A Chip Kit, 16 rxns PN‐1000009, Chromium i7 Multiplex Kit, 96 rxns PN‐120262. Twenty‐eight basepair of cell barcode, and UMI sequences and 91 bp RNA reads were generated with Illumina NovaSeq 6000 at CMG of Indiana University School of Medicine. CellRanger 3.0.2 was utilized to process the raw sequence data generated. Briefly, CellRanger used bcl2fastq to demultiplex raw base sequence calls generated from the sequencer into sample specific FASTQ files. The FASTQ files were then aligned to the rat reference genome with RNAseq aligner STAR. The aligned reads were traced back to individual cells and the gene expression level of individual genes were quantified based on the number of UMIs (unique molecular indices) detected in each cell. The filtered feature‐cell barcode matrices generated by CellRanger were used for further analysis. A representative sample from each condition (similar trends in differentially expressed genes [DEG] were observed in replicates) that were run at the same time to avoid batch effects was selected based on sequencing depth to be used for further downstream analysis as described below.

scRNAseq analysis was performed with R version 4.0.5 (Shake and Throw) and Seurat version 4.0.3. Three biological replicates of each experimental cohort were integrated into a summative object representing the cohort and all four cohort objects were merged into a final Seurat object. Quality control cutoffs were: mitochondrial % <10, and total RNA counts >5000 and <200. Uniform Manifold Approximation and Projection (UMAP) was produced using the first 50 principal components. Differential gene expression (DGE) analysis for each cell cluster was performed with Wilcoxon rank sum test. Differentially expressed genes are those whose expression between two groups differed by at least 0.20 log fold change and were expressed in at least 10% of total cells. Gene set enrichment analysis (GSEA) for each cell population was performed on only significantly differentially expressed genes where adjusted *p* < 0.05. Gene rat annotations were pulled from the Org database. Plots were produced with ggplot2, enrichplot, fmsb, and tidyverse.

### 2.5 Statistics

All statistics were done using Prism software version 9 (GraphPad). Analysis of variance with Šidák's multiple comparison correction was used for the comparison of three or more groups unless otherwise stated. Wilcoxon rank sum test with false discovery rate correction was used for DGE analysis. In considering statistically significant gene expression of gene expression, we used statistics generated from pairwise comparisons within the OVA‐challenged or PBS‐challenged groups (Table S3). We were less stringent and allowed for evaluation of genes that have only a significant unadjusted *p* value as they still potentially demonstrate a true difference between populations.

### 2.6 Multiplex

Cells from splenocytes collected from male and female CDH and RA‐conditioned rats were stimulated with OVA or a vehicle for 24 h. Supernatants were collected and processed for Multiplex analysis. BALF fluid was collected from CDH and RA‐conditioned rats sensitized and challenged with OVA and processed for multiplex analysis.

### 2.7 Airway reactivity

Anesthetized (Ketamine/Xylazine), tracheotomized, and paralyzed (Pancuronium) animals were mechanically ventilated (Flexivent—murine model). Respiratory resistance was assessed with the Flexivent at baseline and then with increased concentrations of inhaled acetylcholine.

## 3 RESULTS

### 3.1 Asthma phenotyping in rats conditioned under chronic hypoxia versus normoxia

**FIGURE 1:**
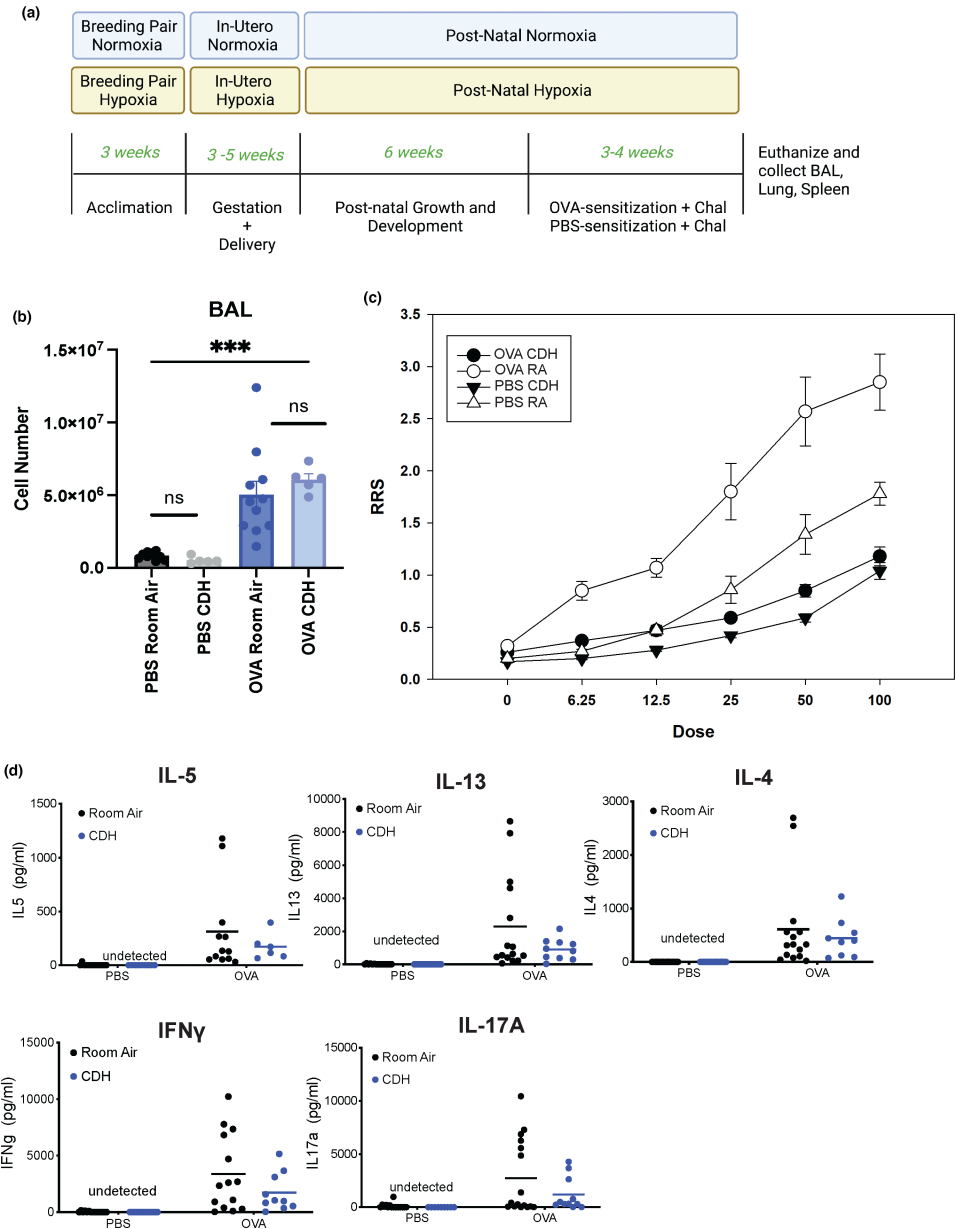
Cytokine production and respiratory resistance in CDH versus room air experienced rats challenged with OVA or PBS. (a) Schematic showing the timeline and outcomes within the congenital developmental hypoxia model. (b) Cell numbers from BAL samples taken from male and female rats at the end of the CDH model. *N* = 5–20. ***p < 0.001. *N* = 5–11, one‐way ANOVA with Tukey's post hoc test for multiple comparisons. (c) Airway reactivity dose response curves generated from male and female rats at the end of the CDH or vehicle sensitization and challenge course. Increasing resistance of respiratory system (RRS) with increasing acetylcholine doses analyzed with repeated ANOVA using log transformed RRS. OVA treatment resulted in a greater response compared to PBS (*p* < 0.0001), and RA had a greater response compared to CDH (*p* < 0.0001). (d) Multiplex analysis of supernatants from OVA‐stimulated splenocytes from CDH versus room air‐conditioned male and female rats sensitized with OVA or PBS. *N* = 10–14. ANOVA, analysis of variance; CDH, chronic developmental hypoxia; OVA, ovalbumin; PBS, phosphate‐buffered saline; RA, room air.

We utilized a model of CDH (Figure 1a) to assess the effect of CDH on the OVA‐induced asthma phenotype. The multiple phenotypes we assessed in the four groups are in Figure 1. OVA sensitization and challenge produced an increase in lung inflammation relative to PBS with increased cellularity, which did not differ between CDH and RA rats (Figure 1b). There was also an increase in airway reactivity following OVA challenge as compared to PBS challenge, however, CDH significantly decreases airway reactivity in both OVA‐ and PBS‐challenged groups (Figure 1c). We then performed a multiplex protein assay for cytokines (IL‐4, IL‐5, IL‐13, IL‐17A, IFNγ) associated with allergic airway inflammation. We found that OVA‐challenged animals showed increased cytokine production from ex vivo OVA‐stimulated (250 μg/ml) splenocytes (Figure 1d). Although there were no differences between the CDH and RA groups, there was a tendency for the CDH group to have lower levels for all cytokines, as well as a more homogenous response within the group compared to RA animals. We additionally collected BALF from all rats but did not see significant differences in BALF cytokine concentrations between CDH and RA rats (Figure S1). These results suggest that CDH had a modest effect on immune sensitization and recruitment of immune cells to the lung but a significant effect on airway hyperreactivity.

### 3.2 scRNAseq overview of cell populations altered by chronic developmental hypoxia and OVA challenge

**FIGURE 2:**
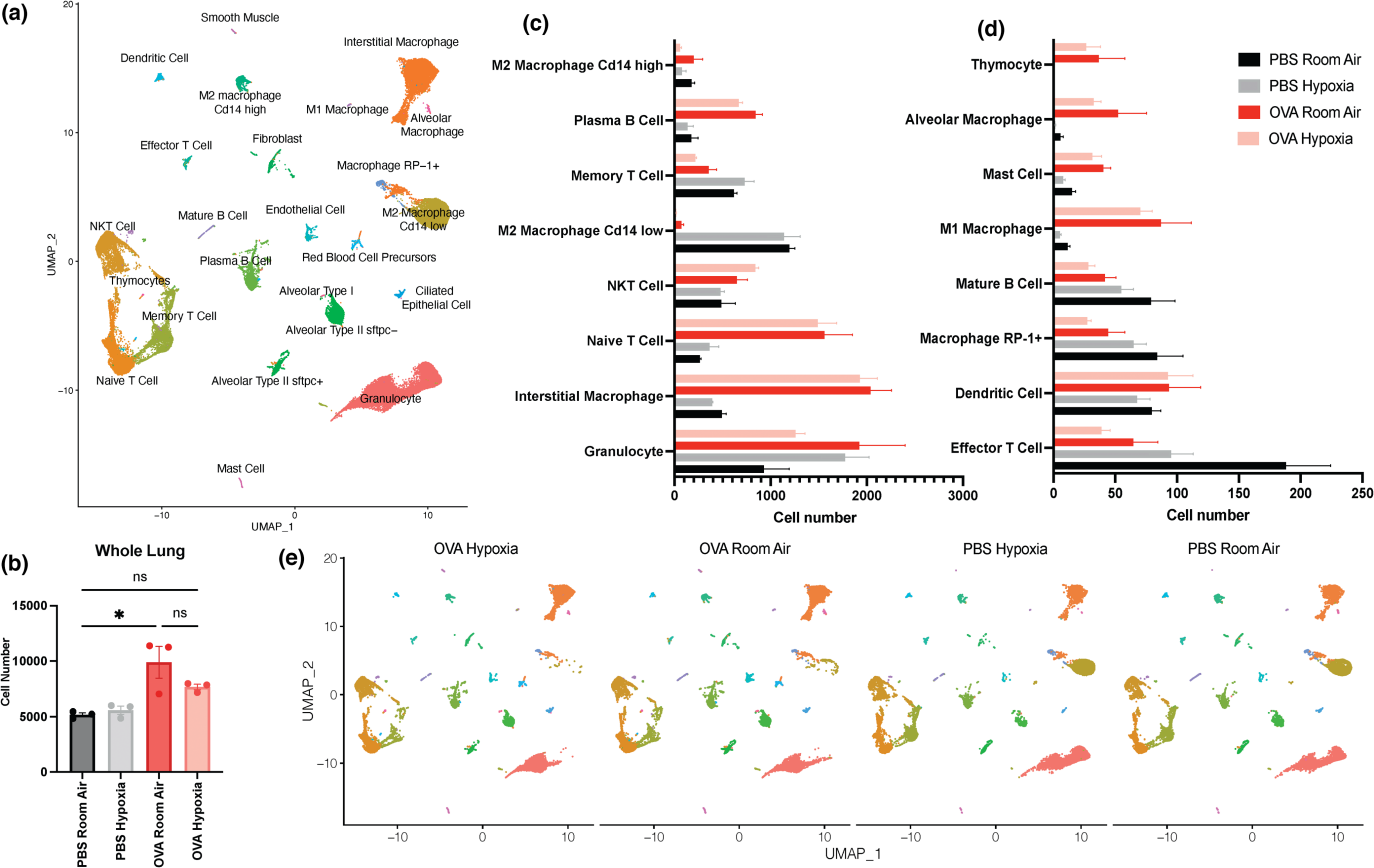
Cell populations from lungs of CDH versus room air‐conditioned male rats sensitized and challenged with PBS or OVA. (a) Combined UMAP of all samples: PBS room air, PBS CDH, OVA room air, OVA CDH with three biological samples each. (b) Total lung cell numbers from the scRNA‐seq datasets. *N* = 3, *p < 0.05. One‐way ANOVA with Tukey's post hoc multiple comparisons test. (c,d) Total cell numbers of lung immune cell populations within each scRNA‐seq experimental condition. *N* = 3. One‐way ANOVA with Tukey's post hoc multiple comparisons test. (e) Deconvoluted UMAP plots displaying each experimental condition. Each plot is a combination of three biological replicates. UMAP, Uniform Manifold Approximation and Projection; ANOVA, analysis of variance; CDH, chronic developmental hypoxia; OVA, ovalbumin; PBS, phosphate‐buffered saline.

We further investigated the potential effects of CDH in this asthma model by performing single‐cell RNA seq analysis of three animals from each of the four groups. Total lung cell numbers included in scRNA‐sequencing from each sample are in Figure 2b. OVA treatment significantly increased lung cellularity compared to PBS treatment. However, CDH in conjunction with OVA treatment showed an intermediate phenotype where cell counts were not significantly different from either PBS‐treated conditions or from the RA OVA condition. The three scRNA‐seq datasets for each experimental condition were merged into a representative dataset for each of the four experimental conditions and the data from the four representative groups was then concatenated into a final comprehensive object for downstream analysis. Unsupervised clustering based on cell‐specific expression of genes produced 24 distinct populations consisting of resident and infiltrating immune cells and lung structural cells (Figure 2a,c,d; Figure S2). We also saw that OVA‐challenged animals have increased specific immune cell populations such as mast cells, interstitial macrophages, plasma B cells, and T cells. The lung structural cell compartment was additionally increased in response to OVA treatment (Figure S2). Also, OVA‐challenged groups had a cell cluster that was not present in either of the PBS‐challenged groups that we identified as red blood cell precursors due to the prominent expression of hemoglobin transcripts (*Hbaa1*, *Hbaa2*, *Hbb*). We next assessed differences in abundance of specific cell populations in response to CDH. While there were no significant differences observed among immune cells (Figure 2c), we do observe trending decreases in the granulocyte, memory T cell, and M2 macrophage compartments in lungs of OVA‐treated rats conditioned to CDH compared to OVA‐treated rats conditioned to RA. In the lungs of PBS‐treated rats, we see trending decreases in the effector T cell population as well as trending increases in the granulocyte, and naïve T cell compartments in response to CDH. In looking at structural cell populations in the lung (Figure S2), we see significant alterations in response to CDH in the fibroblast and ciliated epithelial cell compartments. There is a significant reduction of fibroblasts in response to CDH under both OVA and PBS treatment. In the ciliated epithelial cell compartment, there is a significant reduction in number in response to CDH with PBS treatment, and a trending reduction with OVA treatment. These data confirm induction of allergic lung inflammation in our model of OVA‐induced asthma as well as suggesting that CDH may alter the abundance of immune and structural cell populations within the lung.

### 3.3 DGE between chronic developmental hypoxia and RA experienced cells

**FIGURE 3:**
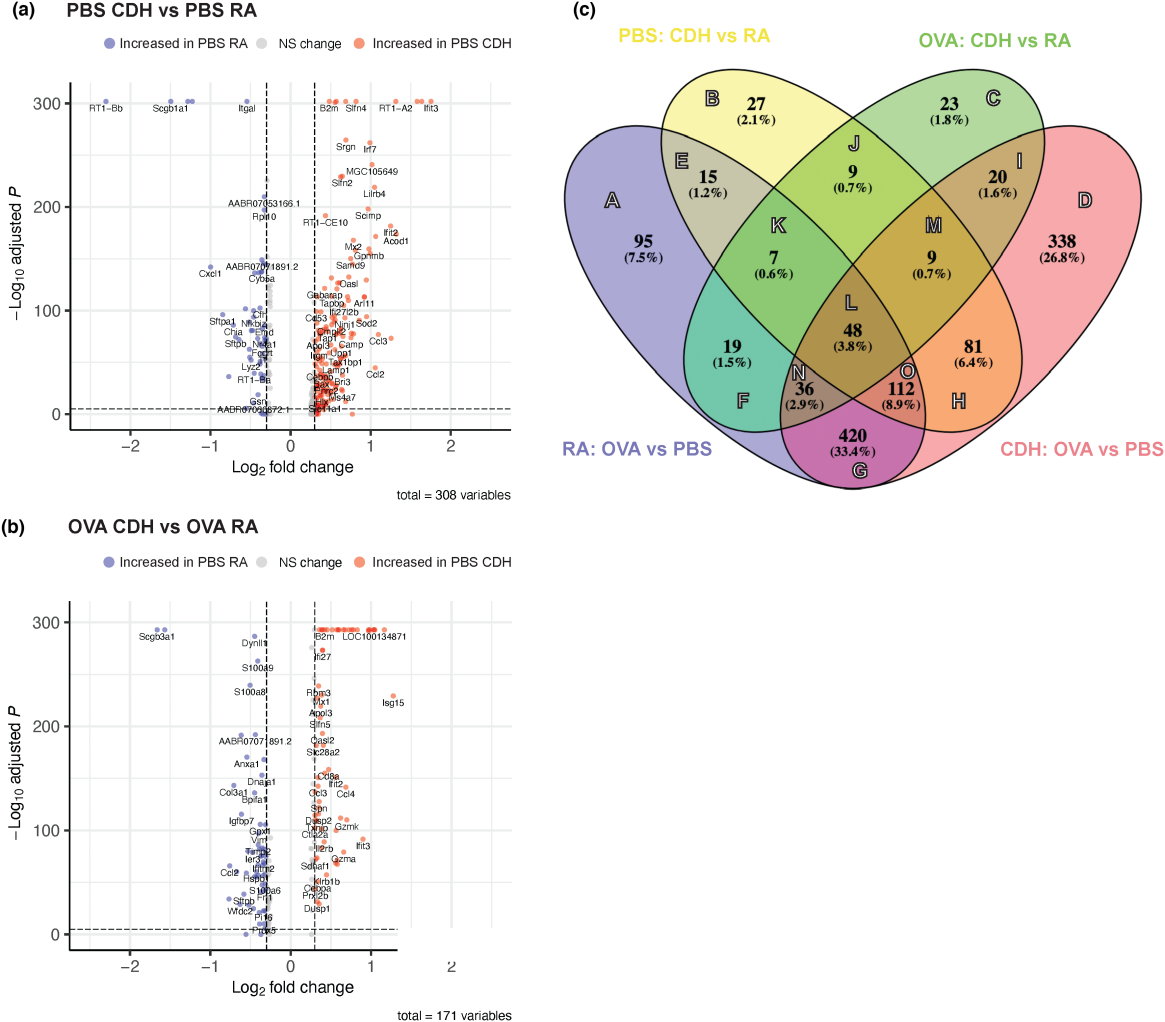
Differential gene expression of total lung cells from rats conditioned to either CDH or room air and sensitized and challenged with either OVA or PBS. (a) Volcano plot showing differentially expressed genes between PBS‐challenged hypoxia versus PBS‐challenged room air experienced rats. Differential gene expression data was generated with Wilcoxon rank sum test with FDR correction. (b) Volcano plot showing differentially expressed genes between OVA‐challenged hypoxia versus OVA‐challenged room air experienced rats. Differential gene expression data was generated with Wilcoxon rank sum test with FDR correction. (c) Venn diagram of a four‐way comparison of differentially expressed genes generated by comparing OVA‐ to PBS‐treated rats from CDH and room air datasets as well as those generated by comparing CDH to room air‐conditioned rats from OVA and PBS datasets. CDH, chronic developmental hypoxia; FDR, false discovery rate; OVA, ovalbumin; PBS, phosphate‐buffered saline.

We next assessed if CDH alters gene expression in immune and structural lung cells by performing DGE analysis between CDH and RA datasets. Under both OVA and PBS treatment conditions, we see a significant increase in genes associated with class I and class II antigen presentation in response to CDH (Figure 3a,b). Additionally, we see significant differential expression of secretoglobulins with *Scgb1a1* being decreased in the PBS‐treated samples and *Scgb3a1* being decreased in the OVA‐treated samples in response to CDH (Figure 3a,b). We then compared DEG lists generated from CDH versus RA conditions and those between OVA versus PBS challenge (Figure 3c). The 48 shared genes between all comparisons likely represent the set of genes involved in the cellular stress response and are induced in response to both CDH and OVA treatment. Of note, we see that while there are 420 shared differentially expressed genes between OVA and PBS treatment, only 95 are unique to cells from the RA condition whereas 338 are unique to those derived from CDH conditions which suggests that, although there is a common response to OVA‐stimulation, CDH impacts this response. The top genes (up to 50) for each Venn diagram section are displayed in Table S1. These data show that CDH significantly alters gene expression in both OVA‐ and PBS‐treated rat lungs, and, thus, that the response to CDH is coordinated by and affects a large number of genes, many of which are specific to the context in which CDH is assessed (i.e., OVA vs. PBS challenge).

### 3.4 Pathway analysis shows putative functional differences in response to chronic developmental hypoxia exposure

**FIGURE 4:**
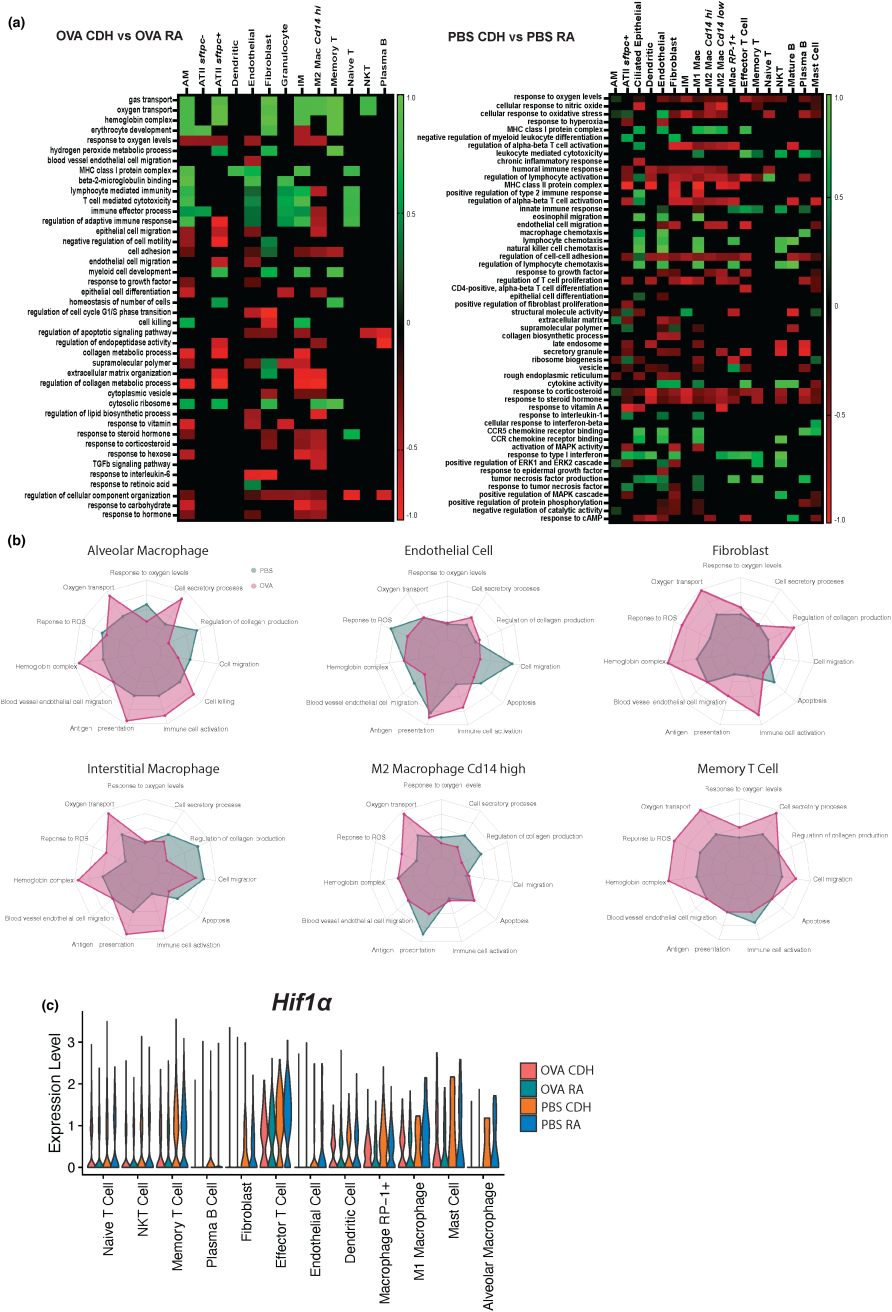
Gene set enrichment pathway analysis between cells from CDH and room air‐conditioned rats sensitized and challenged with either OVA or PBS. (a) Heatmap of differentially enriched pathways in cell populations of interest between hypoxia and room air experienced rats sensitized and challenged with either PBS or OVA. (b) Radar plots showing summarized pathway categories and relative enrichment of pathways of hypoxia versus room air experienced rats from PBS and OVA sensitized and challenged cohorts. (c) Violin plot showing distribution of *Hif1a* transcript expression across the experimental conditions. OVA, ovalbumin; PBS, phosphate‐buffered saline.

Since CDH significantly altered gene expression in both OVA‐ and PBS‐treated rat lungs, we next wanted to evaluate the pathways to which the differentially expressed genes belong. To do so, we selected all significantly (*p* < 0.05) differentially expressed genes and then utilized GSEA to identify affected pathways and their differential enrichment within cell populations in CDH versus RA rats (Figure 4a,b).

Comparing OVA sensitized and challenged rats in CDH versus RA conditions, there is increased induction of pathways related to gas transport in several cell populations in CDH conditions (Figure 4a,b). In addition, pathways related to cytotoxicity and immune cell activation are upregulated in response to CDH. Motility and chemotaxis pathway enrichment are also increased in response to CDH under OVA treatment. Since many of the effects of hypoxia are mediated through HIF‐1α, we looked at transcripts of *Hif1a* across the cell populations (Figure 4c). Only *Cd14*‐low M2 macrophages have significantly reduced expression of *Hif1a* in response to CDH. However, mast cells did demonstrate a trending increase in *Hif1a* in response to CDH.

In the PBS sensitized and challenged rats, we observed that the majority of pathways related to cytotoxicity and immune cell activation are downregulated in response to CDH (Figure 4a,b). Several cell populations also show decreased enrichment in motility and chemotaxis‐related pathways in response to CDH. When examining *Hif1a* expression, we see that it is significantly increased in *Cd14*‐low M2 macrophages and *RP‐1+* macrophages in response to CDH. The increase in *Hif1a* expression in plasma cells was not significant. The remaining cell populations demonstrated similar measures of *Hif1a* expression in response to CDH.

Cells from both the PBS‐ and OVA‐challenged rats demonstrate decreased response to oxygen levels across the majority of cell populations. Also, irrespective of OVA or PBS challenge, we see a decrease in pathways related to organ tissue structure such as the extracellular matrix and collagen production in response to CDH. With respect to specific signaling pathways, we observed that CDH decreased expression of genes involved in the response to steroid hormones. Additional radar plots of specific signaling pathways are shown in Figure S3. We observe that *Hif1a* is most highly expressed in T cells, macrophages, mast cells, fibroblasts, endothelial cells, and dendritic cells. There is an overall decreased expression of *Hif1a* compared to PBS controls (Figure 4c).

Here, we have broadly demonstrated that CDH induces significant differential enrichment of various functional pathways (primarily those related to oxygen transport, ECM organization, and chemotaxis) and induces significant alterations in *Hif1a* expression with PBS and OVA treatment in immune and structural rat lung populations.

### 3.5 Characterization of cell activation and exhaustion states

We next evaluated the mechanism by which these immune cell populations may attenuate the allergic response. Since hypoxia has been shown to dampen the immune response in certain contexts by promoting immune cell exhaustion (Koh et al., 2015; Liu et al., 2020; Westendorf et al., 2017), we wondered if our model of CDH would also decrease activation markers and increase exhaustion markers in CDH‐experienced rats compared to normoxia‐experienced rats.

**FIGURE 5:**
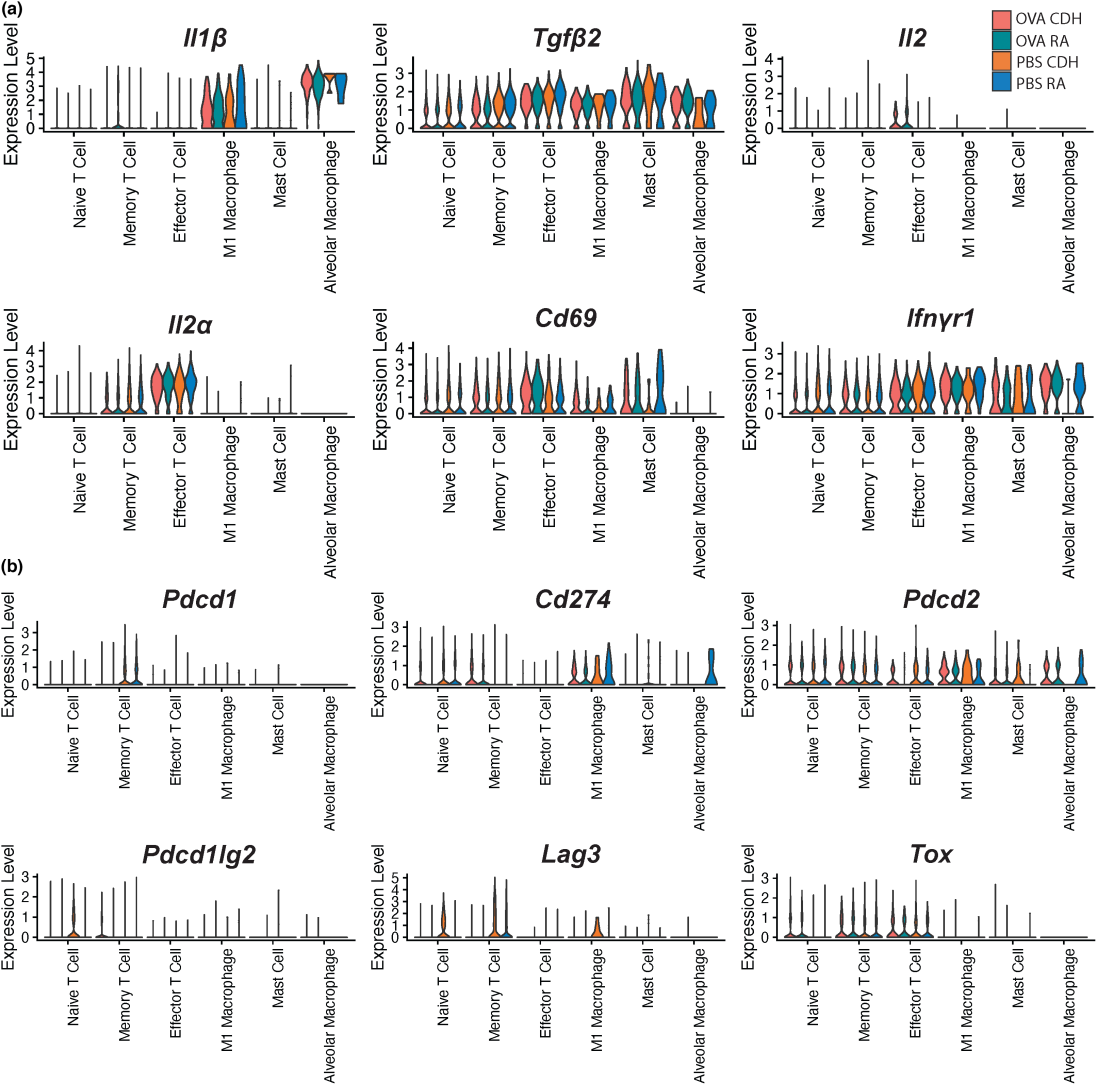
Activation and exhaustion markers across immune cell populations. (a) Violin plots of activation marker transcript expression in immune cell populations from CDH and room air‐conditioned rats sensitized and challenged with either OVA or PBS. (b) Violin plots of exhaustion marker transcript expression in immune cell populations from CDH and room air‐conditioned rats sensitized and challenged with either OVA or PBS. OVA, ovalbumin; PBS, phosphate‐buffered saline.

We examined inflammatory and activation markers (Figure 5a) to determine whether there was an effect of CDH versus RA on gene expression in OVA‐sensitized and ‐challenged rats. *Il1b*, a common inflammatory marker and *Tgfb*, which has been shown to be a critical mediator in lung injury and promote inflammatory lung disease (Pittet et al., 2001; Thornburg et al., 2010) were not significantly altered by CDH. The genes encoding IL‐2 (*Il2*) and its receptor CD25 (*Il2ra*) are primarily expressed by T cell populations but were not altered by CDH. *Cd69*, an early activation marker, is increased significantly in M1 macrophages, memory T cells, and naïve T cells in response to CDH. *Ifngr1* expression remains intact in alveolar macrophages from OVA‐treated rats in CDH. We also evaluated the effect of CDH upon exhaustion markers (Figure 5b) across immune cell populations. Note that any mention of significance is based on computed DEG results (Table S2). There were no significant differences in the panel of exhaustion markers in response to CDH under OVA challenge conditions.

We also evaluated T cell‐associated activation and exhaustion markers in the PBS‐treated cohort. We found a decrease in *Ifngr1* expression in hypoxic alveolar macrophages that trended toward significance. *Il1b* and *Tgfb1* are similarly expressed between the hypoxic and normoxic conditions. *Cd69* is significantly decreased in mast cells in response to CDH. The exhaustion marker *Pdcd1* (PD‐1) was only notably expressed in the memory T cell population. *Cd274* (PD‐L1) produces the ligand for PD‐1 and expression is modestly decreased in M1 and alveolar macrophages. *Pdcd2* (PD‐2) is another exhaustion marker whose expression is increased significantly in mast cells from hypoxic rats. We also see a drop in response to CDH in the alveolar macrophage population. *Pdcdlg2* (PD‐L2) was only notably expressed on naïve T cells and was not significantly altered in response to CDH. *Lag3*, another exhaustion marker, is significantly increased in naïve T cells and memory T cells in response to CDH during PBS challenge. *Tox* was preferentially expressed on T cell populations and did not demonstrate significant alterations in response to CDH.

Lastly, we evaluated these markers across both OVA‐ and PBS‐treated animals, these data indicate alteration of expression of the prominent activation marker, *Cd69*, across several immune cell populations in response to CDH with both PBS and OVA treatment. We also see that CDH increases T cell exhaustion marker expression with PBS treatment, although this change is lost when rats are challenged with OVA.

Taken together, we see alterations in activation and exhaustion markers in various immune cell populations in response to CDH.

### 3.6 Characterization of airway inflammation associated markers

We next assessed the transcript expression of immune cell secretory products as cytokines and secretoglobulins produced by different immune cell populations are critical for mediating the allergic response and creating the inflammatory milieu. Note that any mention of significance is based on computed DEG results (Table S2).

**FIGURE 6:**
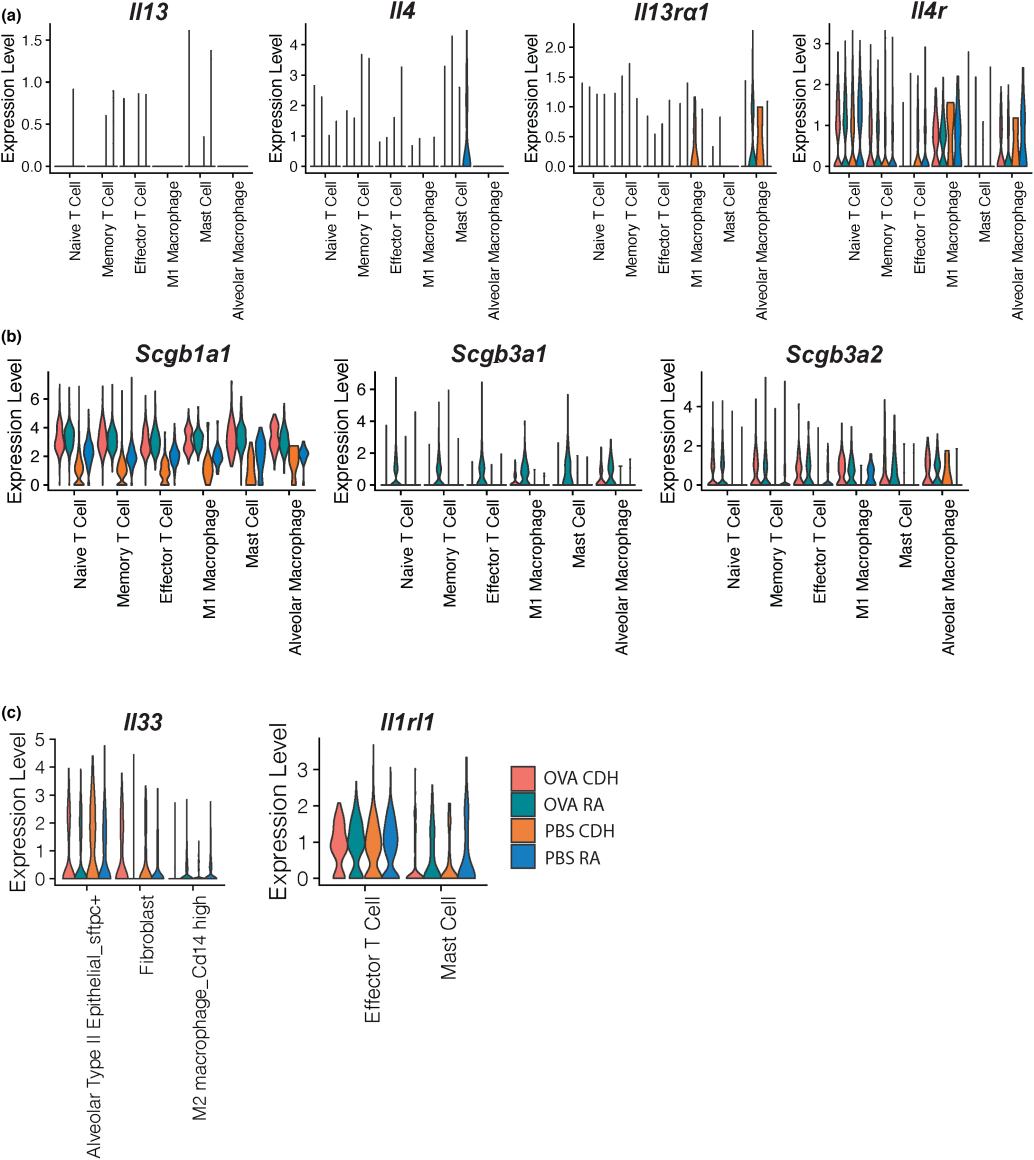
Inflammatory marker expression across immune and structural cell populations. (a) Violin plots of Th2 associated transcript expression in immune cell populations from CDH and room air‐conditioned rats sensitized and challenged with either OVA or PBS. (b) Violin plots of secretoglobulin transcript expression in immune cell populations from CDH and room air‐conditioned rats sensitized and challenged with either OVA or PBS. (c) Violin plots of *Il33* and cognate receptor *Il1rl1* transcript expression in structural and immune cells from CDH and room air‐conditioned rats sensitized and challenged with either OVA or PBS. CDH, chronic developmental hypoxia; OVA, ovalbumin; PBS, phosphate‐buffered saline.

We compared the response to CDH versus RA in OVA‐treated rats. Expression of *Il13* and *Il4* was only observed in a few cells. Expression of *Il13ra* and *Il4ra* also showed minimal differences in response to CDH (Figure 6a). With respect to *Scgb1a1* only memory T cells, mast cells, and alveolar macrophages demonstrated a significant CDH‐induced increase in expression with OVA treatment (Figure 6b). *Scgb3a1* had significantly decreased expression in all cell populations in response to CDH. *Scgb3a2* showed overall consistent expression between hypoxic and normoxic rats. *Il33* expression was significantly increased in fibroblasts in response to CDH. However, *Il1rl1* expression was not significantly altered in response to CDH (Figure 6c).

In comparing PBS‐treated rats in CDH versus RA conditions, expression of *Il13* and *Il4* was modest with only mast cells expressing detectable amounts of *Il4* transcripts under normoxic conditions. *Il13ra1* and *Il4ra* expression were not significantly altered (Figure 6a). Alterations in secretoglobulin expression correspond to the overall inflammatory environment. *Scgb1a1* expression is significantly decreased in all displayed cell populations in response to CDH, suggesting a decreased inflammatory phenotype in the CDH rats. Minimal *Scgb3a1* expression was seen in PBS‐treated rats, although CDH did induce a significant decrease in naïve T cells. *Scgb3a2* was significantly decreased in memory and effector T cells, M1 macrophages, and increased in alveolar macrophages in response to CDH (Figure 6b). *Il33* was significantly increased in *Sftpc +* alveolar type II epithelial cells. Expression of *Il1rl1* displayed a trending decrease in response to CDH but was not significant (Figure 6c).

Taken together, the overall decrease in inflammation‐induced secretoglobulin expression, particularly of *Scgb3a1* suggests that the inflammatory environment was significantly diminished in response to CDH.

## 4 DISCUSSION

This study evaluated whether CDH would suppress the immune response to allergic lung inflammation, as well as evaluate the effect of CDH in the control non‐allergic state. CDH significantly altered gene expression and multiple molecular pathways in both OVA‐treated and PBS‐treated rats. Some trends in CDH‐mediated alteration were the same between OVA‐treated and PBS‐treated rats and some were unique to either treatment group. CDH altered activation and exhaustion markers across several immune cell populations, and also impacted secretoglobulin expression in both conditions. Collectively, our findings suggest that CDH alters various immune cell populations, which could affect responses to various stimuli; however, we did not detect a large impact of CDH upon the pulmonary response specifically due to allergic inflammation.

We demonstrated fidelity of our model of OVA‐induced asthma as evidenced by increased airway hyperreactivity, cellularity, and Th2 cytokine production. While CDH significantly decreased airway hyperreactivity and produced a trending decrease in cytokine production, these differences were common to both OVA‐treated and PBS‐treated rats. Additionally, while airway hyperreactivity was decreased, our scRNA‐seq count data for smooth muscle cells were not significantly different in response to CDH in either OVA‐treated or PBS‐treated rats. While no studies have investigated the role of congenital developmental hypoxia on airway smooth muscle, several studies have shown that chronic hypoxia plays a role in airway smooth muscle growth (Lee & Kang, 2019; Minamino et al., 2001; Xu et al., 2021). Although we were interested in assessing the differentially expressed genes and enriched pathways of smooth muscle cells to evaluate gene sets related to growth, there was an insufficient number of cells for robust analysis in our current dataset. We also had an insufficient number of smooth muscle cells to distinguish between vascular and airway smooth muscle. We plan to continue exploring the role of smooth muscle cells in CDH in future studies.

While the physiological response to CDH was similar between OVA‐treated and PBS‐treated rats, our scRNA‐seq data demonstrated both common and unique responses to CDH in the context of OVA treatment or PBS treatment in various lung cell populations as evidenced by sets of differentially expressed genes and differentially enriched pathways. However, among the various parameters assessed, very little pointed to a differential Th2 inflammatory response due to CDH exposure. However, CDH did affect the inflammatory response in other notable ways:

We saw that there were both common and unique significantly differentially expressed genes in OVA‐treated and PBS‐treated rats in response to CDH. We saw that there was a significant increase in MHC class II expression in response to CDH which is consistent with previous reports studying post‐natal hypoxia exposure (Aiello et al., 2013; Kajiwara et al., 2016). Also of note is that the numbers of differentially expressed genes pulled from scRNA‐sequencing was far lower than the numbers obtained from bulk RNA sequencing, reflecting the increased depth of sequencing from bulk RNA sequencing. The relatively shallow sequencing afforded by scRNAseq is a limitation of the study and may contribute to the lack of allergic‐specific responses found in response to CDH.

We also saw differences among the differentially enriched pathways in response to CDH in both OVA‐treated and PBS‐treated rats. Pathways related to the oxygen transport were downregulated in response to hypoxia in several cell populations during PBS challenge but upregulated during OVA challenge. One potential explanation for these divergent responses is that, during OVA challenge, there is an increase in oxygen transport that is not seen in the PBS challenge. This is likely due to the sudden increased need for oxygen due to the airway inflammation that perhaps provides greater compensation for the underlying hypoxic state. These differential responses are reflective of the large number of differentially expressed genes that are unique to the OVA challenge (Figure 3d). Additionally, we see that increased enrichment of pathways related to hemoglobin protein complexes in cells from OVA‐treated rats as compared to PBS‐treated rats. Expression of hemoglobin in non‐erythroid lineages such as macrophages, epithelial cells, and mesangial cells has been previously reported to mitigate oxidative stress (Liu et al., 1999; Nishi et al., 2008; Saha et al., 2014) and their expression may serve a similar function in our models as previous studies have shown that hypoxia can increase ROS production (Azimi et al., 2017; Bell et al., 2007). The differential enrichment of pathways related to ECM thickness and collagen production in response to CDH is consistent with the published ability of hypoxia to decrease ECM thickness and collagen production. McKay et al. showed that acute hypoxia decreases collagen secretion in human corneal fibroblasts, and Bentovim et al. reported that HIF‐1α acts as a central regulator of collagen production in chondrocytes in the hypoxic growth plate (Bentovim et al., 2012; McKay et al., 2017). The differential enrichment of pathways related to the hormone response by cells in response to CDH is consistent with previous studies showing that this effect is mediated through HIF‐1α by directly regulating steroidogenesis in the adrenal gland (Chang et al., 2010; Watts et al., 2021).

In our assessment of activation and exhaustion markers in T cells and macrophages, we saw trends in expression of several markers although most were not significant. This indicates that while CDH impacts RNA expression of activation and exhaustion markers in T cells and macrophages, the effect of these alterations likely does not contribute significantly to the attenuation of allergic inflammation.

In our assessment of immune cell inflammatory markers, we found little expression of classic inflammatory cytokines. We had additionally assayed for *Il9*, *Il5*, *Il1a*, *Il17a/f*, but gene expression of these cytokines was below detection. PBS‐treated M1 macrophages had increased *Il13ra* expression in response to CDH which may alter their cytokine responsiveness. Secretoglobulin expression was also significantly altered in response to CDH. We observed that CDH decreased *Scgb1a1* expression in PBS‐treated rats and decreased *Scgb3a1* in OVA‐treated rats globally. *Scgb1a1*, primarily studied in the context of the airway epithelium, has previously been shown to have an immune‐suppressive effect and is typically increased under inflammatory stress (Xu et al., 2020; Zhu et al., 2019). *Scgb3a1* is less well studied but has been shown to play some roles in inflammation. A report by Raffay et al. showed that neonatal hyperoxia followed by RA recovery was associated with a decrease in SCGB3A1 protein (Raffay et al., 2013). Yamada et al. (2005, 2009) showed that SGB3A1 expression is induced by IL‐4 and IL‐3 which are classic Th2 cytokines that are critical in allergic airway disease.

Previously, few studies have investigated the role of congenital developmental hypoxia in the development and function of the lung in the context of allergic airway disease. In this study, we have documented alterations in gene expression caused by congenital developmental hypoxia in murine lung immune cell populations and transcriptomic profiles in the presence or absence of allergic lung inflammation.

## AUTHOR CONTRIBUTIONS

Michelle Chu and Hongyu Gao performed and analyzed scRNA‐seq studies. Huanling Gao and Patricia Esparza sensitized and challenged rats and performed airway reactivity testing. Abigail Pajulas, Jocelyn Wang, and Rakshin Kharwadkar performed immune cell isolations, cultures, and analysis of cytokines. Mark H. Kaplan and Robert S. Tepper conceived and planned the studies. Michelle Chu, Mark H. Kaplan, and Robert S. Tepper wrote the paper.

## ETHICAL STATEMENT

Studies involving animals in this report were approved by the Institutional Animal Care and Use Committee.

## Supporting information


Figure S1.
Figure S2.
Figure S3.

Table S1.
Table S2.
Table S3.
