# Supplementary material for: Chronic developmental hypoxia alters rat lung immune cell transcriptomes during allergic airway inflammation
Source: Physiol Rep. 2023 Feb 7;11(3):e15600. doi: 10.14814/phy2.15600 (PMC9904961; doi:10.14814/phy2.15600)
Supplement: Supplementary file 1 — Figure S1. Figure S2. Figure S3. [file PHY2-11-e15600-s002.docx]

Chronic developmental hypoxia alters rat lung immune cell transcriptomes during allergic airway inflammation

Michelle Chu^1^, Huanling Gao^2^, Patricia Esparza^2^, Abigail Pajulas^1^, Jocelyn Wang^1^, Rakshin Kharwadkar^1^, Hongyu Gao^3^, Mark H. Kaplan^1,2*^, Robert S. Tepper^2*^

**Supplementary Figures**

Supplementary Figure 1: Multiplex analysis of BALF

Multiplex analysis of cytokines in BALF from CDH and room air-conditioned rats sensitized and challenged with OVA. N = 5-7, Student’s t test.

Supplementary Figure 2: Primary cluster markers

(A) Heatmap showing the top five markers per cluster used for their respective identification.

(B) Numbers of lung structural cells. N = 3, **p < 0*.05, ***p* < 0.01. One-way ANOVA with Tukey’s post hoc multiple comparisons test.

Supplementary Figure 3: GSEA of signaling pathways in immune cell populations

Radar plots showing differential enrichment of signaling pathways in immune cell populations in CDH vs room air conditioned rats sensitized and challenged with either PBS or OVA.
