# Supplementary material for: Chronic developmental hypoxia alters rat lung immune cell transcriptomes during allergic airway inflammation
Source: Physiol Rep. 2023 Feb 7;11(3):e15600. doi: 10.14814/phy2.15600 (PMC9904961; doi:10.14814/phy2.15600)
Supplement: Supplementary file 2 — Table S1. Table S2. Table S3. [file PHY2-11-e15600-s001.docx]

**A B C D E F G H**

**I J K L M N O**

Supplementary Table 1: Differentially expressed gene lists

Differentially expressed genes corresponding to the same-colored and same-numbered sector of the Venn diagram from Figure 2C.

Supplementary Table 2

| **GSEA Pathway** | **Genes** |
| --- | --- |
| Gas Transport | Cygb, Ipcef1, Hba1, Hba2, Hbm, Hbb, Hbd, Hbe1, Hbg1, Hbg2, Hbq1, Hbz, Aqp1, Aqp4, Aqp5, Aqp6, Mb, Myc, Ngb, Rhag, Bpgm, Ca2 |
| Oxygen Transport | Cygb, Ipcef1, Hba1, Hba2, Hbm, Hbb, Hbd, Hbe1, Hbg1, Hbg2, Hbq1, Hbz, Mb, Myc, Ngb, Bpgm |
| Cellular Response to Nitric Oxide | Hdac5, Bcl2l11, Hcn4, Mir1271, Mir762, Pqbp1, Actr2, Nampt, Akap9, Cdk2, Cdk4, Cdk5, Flot1, Rragb, Tlr6, Klf2, Nod1, Rapgef3, Prmt5, Baiap2 |
| Response to Hyperoxia | Atg7, Foxo1, Nox1, Fas, Cav1 |
| Hemoglobin Complex | Cyb5r3, Hba1, Hba2, Hbm, Hbb, Hbd, Hbe1, Hbg1, Hbg2, Hbq1, Hbz, Ahsp |
| Erythrocyte Development | Sh2b3, Maea, Klf2, Cited2, Fam210b, Epb42, Dmtn, Epo, Ercc2, Alas1, Alas2, Adgrf5, Jmjd6, Abcb10, Brd1, G6pd, Trim58, Gata1, Flvcr1, Tmod3 |
| Response to Oxygen Levels | Adam8, Cdkn1b, ENSG00000274276, Stub1, Cited2, Ndrg1, Acaa2, Hyou1, Atg7, Usp19, Egln2, Phb2, Ucn3, Card16, Vasn, Clca1, Plk3, Cpeb2 |
| Hydrogen Peroxide Metabolic Process | Hdac6, Prdx4, Mtco2p12, Noxa1, Prdx3, Park7, Noxo1, Pxdnl, Ctns, Cyba, Cybb, Cyp1a1, Cyp1a2, Fyn, Mpv17l, Prdx5, Nox1, Gpx1, Gpx3, Hba1 |
| Blood Vessel Endothelial Cell Migration | Akt3, Mir939, Hdac5, Cdh5, Mir2355, Cib1, Plk2, Pdcd10, Nus1, Cln3, Mapk14, Csnk2b, Cyp1b1, Amot, Clec14a, Spred1, Gadd45a, Agtr2, Efna1, Efnb2 |
| MHC Class I Protein Complex | Hfe, Hla-a, Hla-b, Hla-c, Hla-e, Hla-f, Hla-g, Hla-h, Mr1, B2m |
| Beta-2-Microglobulin Binding | Mica, Fcgrt, Hfe, Hla-a, Hla-e, Hla-f, Hla-h, Mr1, Cd1d |
| Lymphocyte Mediated Immunity | C17orf99, Parp3, Igll5, Klrf2, Mica, Klrc4-Klrk1, Rasgrp1, Cd96, Ighv2-70d, Ighv1-69d, Tcirg1, Tubb4b, Btn3a2, Mad2l2, Batf, Cebpg, Cd226, Masp2, Ctsc |
| T Cell Mediated Cytotoxicity | Mica, Ctsc, Lilrb1, Ripk3, Il23r, Ctsh, Ager, Emp2, Fcgr2b, Cadm1, Gzmm, Nckap1l, Hla-a, Hla-b, Hla-dra, Hla-drb1, Hla-e, Hla-f, Hla-g, Hla-h |
| Eosinophil Migration | Adam8, Ccl26, Cd300a, Dapk2, Hrh1, Il4, Lgals3, Ptger4, Ccl1, Ccl2, Ccl3, Ccl4, Ccl5, Ccl7, Ccl8, Ccl11, Ccl13, Ccl21, Ccl24, Cx3cl1, Scg2 |
| Macrophage Chemotaxis | Cmklr1, Csf1, Csf1r, Il34, Tafa4, Cyp19a1, Ddt, Edn2, Ednrb, Ptk2b, Stap1, Mstn, Cxcl17, Lgals3, Mdk, Mif, Mmp2, Cklf, Mapk1, Mapk3 |
| Natural Killer Cell Chemotaxis | Klrc4-Klrk1, Klrk1, Pik3cd, Pik3cg, Ccl2, Ccl3, Ccl4, Ccl5, Ccl7, Xcl1 |
| Immune Effector Process |  |
| Regulation of Adaptive Immune Response | Ada, C17orf99, Parp3, Mad2l2, Batf, Cd226, Tnfsf13b, Lilrb1, Arid5a, Malt1, Lilrb4, Ripk3, Cd160, Dusp10, Shld3, Adcy7, Nlrp3, Csmd3, Clc, Exosc6 |
| Epithelial Cell Migration | Ada, C17orf99, Parp3, Igll5, Klrf2, Mica, Klrc4-Klrk1, Rasgrp1, Cdh17, Cd96, Ighv2-70d, Ighv1-69d, Ighv3-64d, Tcirg1, Tubb4b, Btn3a3, Nod1, Spon2, Vav3, Mad2l2 |
| Regulation of Cell Motility | Arpc3, Actr3, Arpc2, Jmy, Eps8, Nckapil, Enah |
| Cell Adhesion | Ada, Cdh2, Mir675, Siglec14, Cdh3, Mir939, Lmln2, Cldn24, Cd24, Bcl2l11, Sh2b3, Cdh4, Gne, Troap, Cldn34, Cdh5, Cdh6, Dnajb6, Cdh7, Klrc4, Klrk1 |
| Endothelial Cell Migration | Akt3, Mir939, Hdac5, Pdcd6, Cdh5, Mir1908, Mir2355, Cdh13, Cib1, Cxcl13, Gipc1, Plk2, Ptp4a3, Fstl1, Pdcd10, Nus1, Cln3, Aamp, Mapk14, Csnk2b |
| Myeloid Cell Development | Abi1, Sh2b3, Tspan2, Wasf2, Maea, Klf2, Cited2, Cebpb, Lilrb1, Fam210b, Slc9b2, Zfpm1, Ep300, Epb42, Dmtn, Epo, Ercc2, Alas1, Alas2, Evi2b |
| Response to Growth Factor | Hdac6, Pdcd6, Mir1224, Cdh5, Mir1298, Tgfbr3l, Micos10-nbl1, Mir4632, Tob1, Fbxw7-as1, Spry3, Spry1, Spry2, Ramp2, Fstl3, Stub1, Cdkn1c, Cdkn2b, Cited2, Rack1 |
| Epithelial Cell Differentiation | Cdh2, Cdh3, Mir541, Cd24, Cdh5, Ush1c, Fem1b, Enam, Abi2, Dhrs9, Cdk6, Gdf11, Spry1, Cdkn1b, Cdkn2b, Ccno, Klf2, Basp1, Cdsn |
| Homeostasis of Number of Cells | Ada, Cdh2, Akt3, Bcl2l11, Sh2b3, Armcx5-Gprasp2, Trim10, Lpcat3, Cdk6, Maea, Adar, Tcirg1, Ikzf1, Klf2, Cited2, Mertk, Znhit1, Cebpg, Klf1, Tnfsf13b |
| Regulation of Cell Cycle G1/S Phase Transition | Mir892b, Mir873, Ctdsp2, Cdk2, Psme3, Ctdspl, Cdk7, Cdkn1a, Cdk2ap2, Cdkn1b, Cdkn2a, Cdkn2b, Cdkn2c, Cdkn2d, Btn2a2, Gpnmb, Plk2, Fam107a, Chek2, Trex1 |
| Cell Killing | Klrf2, Mica, Klrc4-Klrk1, Rasgrp1, Tubb4b, Cebpg, Gnly, Cd226, Ctsc, Lilrb1, Ripk3, Cd160, Chga, Coro1a, Lyst, Tusc2, Pglyrp3, Slamf6, Dcd, Defb118 |
| CCR5 Chemokine Receptor Binding | Nes, Cnih4, Jak1, Ccl3, Ccl4, Ccl5, Stat1 |
| Positive Regulation of ERK1 and ERK2 Cascade | Apela, Rasgrp1, Spry2, Ramp3, Ccl26, Abca7, Nod1, Gpnmb, Cib1, Fermt2, Chi3l1, Cavin3, Phb2, Chrna7, Tirap, Adcyap1, Ccr1, Ccr7, Crkl, Csfir |
| Regulation of Apoptotic Signaling Pathway | Bcl2l10, C8orf44-sgk3, Ppif, Trap1, Psme3, Cdkn2d, Yap1, Acaa2, Creb3, Hyou1, Ivns1abp, Zmynd11, Park7, Rffl, Clu, Lrrk2, Col2a1, Csf2, Csnk2a1, Csnk2a2 |
| Regulation of Endopeptidase Activity | Lmln2, Jmjd7, Pigk, Usp17l10, Usp17l11, Usp17l12, Usp17l13, Usp17l17, Prss46p, Usp17l18, Usp17l19, Usp17l20, Usp17l21, Usp17l22, Usp17l15, Casp12, Adam8, Cela3a, Adam10, Zmpste24 |
| Collagen Metabolic Process | P3h3, P3h4, Mmp24, Emilin1, Cygb, Mmp21, Col1a1, Col1a2, Col5a1, Adamts14, Cst3, Ccn2, Ctsb, Ctsk, Ctsl, Ctss, Cyp7a1, Retreg3, Tmprss6, Eng |
| Supramolecular Polymer | C19orf71, Hdac6, Kif28p, Krtap25-1, Krtap4-9, Krtap20-4, Krtap22-2, Krtap21-3, Chmp4bp1, Dnajb6, Krtap9-7, Krtap16-1, Krtap9-6, Krtap29-1, Dnmil, Abcc9, Myzap, Kif20a, Dnal4, Lrpprc |
| Extracellular Matrix Organization | Ramp2, Fbln5, Emilin1, Col1a2, Col3a1, Dag1, Agt, Lamb4, Ntng1, Clasp2, Phldb1, Clasp1, Gas6, Gpm6b, Rgcc, Efemp2, Has1, Has2, Has3, Ihh |
| Regulation of Collagen Metabolic Process | Emilin1, Cygb, Cst3, Ccn2, Cyp7a1, Eng, F2, F2r, Fap, Amelx,Rgcc, Hdac2, Ihh, Il6, Inhba, Itga2, Mir145, Mir149, Mir218-1 |
| Cytosolic Ribosome | Rack1, Rpl35, Rpl39l, Rps4y2, Rpl10l, Fau, Rplp0p6, Rpl13a, Rpl36, Rpl7l1, Hba1, Hba2, Apod, Rpsa, Eif2ak4, Rpl10a, Rps27l, Rpl26l1, Zcchc17, Dhx29 |
| Regulation of Lipid Biosynthetic Process | Mir548p, Nr1h3, Lpcat3, Cdk4, Prmt3, Pibf1, Kat5, Sorbs1, Erlin1, Ces1, Avil, Cga, Erlin2, Ddx20, Chp1, Trex1, Apoa5, Clcn2, Fitm2, Adm |
| Response to Vitamin | ENSG00000274276, Cdkn2d, Postn, Col1a1, Cyp1a1, Cyp24a1, Cyp26a1, Cyp27b1, Dnmt3a, Epo, Alad, F5, F7, Tpcn2, Fes, Fkbp1b, Snw1, Folr1, Folr2 |
| Response to Steroid Hormone | Nr2e3, Hdac6, Bcl2l11, Nr1h3, Ptpru, Pdcd7, Cdo1, Yap1, Pgrmc2, Tada3, Carm1, Ncoa2, Ddx17, Txnip Ptges3, Gpr83, Abhd2, Sox30, Fam107a, Egln2 |
| Response to Corticosteroid | Bcl2l11, Ptpru, Pdcd7, Cdo1, Gpr83, Sox30, Fam107a, Ucn3, Fbxo32, Tph2 |
| Response to Interleukin-6 | St3gal6, Yap1, Cebpa, Ptprt, Chi3l1, C1qtnf4, Fer, Fgg, Sbno2, Gfi1, Foxa2, Il6, Il6r, Il6st, Aqp4, Jak1, Jak2, Mirlet7a1, Mirlet7c, Mirlet7e |
| Response to Retinoic Acid | Cdkn2d, Yap1, Trim16, Fzd10, Phb2, Twf2, Ovca2, Col1a1, Osr1, Creb1, Ctsh, Cyp26a1, Brinp1, Asxl1, Abca1, Epha3, Ptk2b, Fgfr2, Adnp2, Snw1 |
| Response to Carbohydrate | Mir320b1, Mir320c1, Mir320d2, Mir320c2, Mir320b2, Mir32d1, Mir320e, Calcrl, Cdkn1b, Rack1, Unc13b, Kat5, Txnip, Slc12a7, Cftr, Gjb6, Adcy5, Kat7, Adcy8, Ucn3 |

Supplementary Table 2: GSEA Gene Lists

Table displaying lists of genes utilized to determine pathway enrichment with GSEA. Maximum of 20 genes are shown.

Supplementary Table 3: Significance scores of differential gene expression data

Table displaying native and adjusted *p* values for two-way comparisons discussed in the text. *p* values were generated with Wilcoxon rank sum test and FDS corrected as noted.
